# Effect of rosuvastatin on the concentration of each fatty acid in the fraction of free fatty acids and total lipids in human plasma: The role of cholesterol homeostasis

**DOI:** 10.1016/j.bbrep.2020.100822

**Published:** 2020-10-02

**Authors:** Cristian I. Ciucanu, Sonia Olariu, Daliborca C. Vlad, Victor Dumitraşcu

**Affiliations:** Pharmacology and Biochemistry Department, Faculty of Medicine, University of Medicine and Pharmacy “Victor Babes” of Timişoara, Piaţa Eftimie Murgu 2, RO-300041, Timişoara, Romania

**Keywords:** Free fatty acid, Total fatty acids, Plasma lipids, Rosuvastatin, Atherosclerosis, Cholesterol homeostasis

## Abstract

Each fatty acid (FA) or class of FAs has a different behavior in the pathologies of atherosclerosis. The aim of this study was to investigate changes in the concentration of each fatty acid in the fraction of free fatty acids (FFAs) and total lipids in human plasma after short-term therapy with rosuvastatin as a cholesterol-lowering statin drug. Six hypercholesterolemic men on a habitual diet were studied in a randomized, double-blind, and crossover process. They received 20 mg rosuvastatin or placebo in random order, each for 4 weeks and after 2 weeks of washout period, they received another medication (placebo or rosuvastatin) for another period of 4 weeks. Rosuvastatin treatment significantly decreased the absolute concentrations of saturated and monounsaturated FAs in the total FAs as well as in FFAs. Long chain polyunsaturated fatty acids with 20 and 22 carbon atoms in the molecule had no significant change in the fraction of FFAs. Rosuvastatin is directly involved in cholesterol biosynthesis and indirectly through cholesterol homeostasis in the biosynthesis of other plasma lipids.

In conclusion, our findings show that rosuvastatin treatment leads to significant changes in the concentration of each fatty acid, except for long-chain polyunsaturated fatty acids in FFAs. Our observations indicate that cholesterol homeostasis through its regulatory mechanisms appears to be the main cause of changes in the concentration of each plasma fatty acid during rosuvastatin treatment. These changes can be a source of beneficial consequences, in addition to lowering low-density lipoprotein cholesterol in cardiovascular diseases.

## Highlights

• Rosuvastatin decreased the concentration of total fatty acids and free fatty acids.
• The change in the concentration of each fatty acid was analyzed.
• Changes in fatty acid concentrations are part of cholesterol homeostasis.
• These changes bring beneficial consequences in cardiovascular diseases.

## 1 Introduction

Statins are a class of drugs with a high applicability in the prevention of cardiovascular diseases due to their efficiency in lowering low-density lipoprotein (LDL) cholesterol, which is consider an important risk factor in atherosclerosis [1]. Atherosclerosis is a complex process initiated with endothelial cell dysfunction, followed by penetration of low-density lipoprotein through the vascular endothelium and oxidation of LDL components [2]. The core of LDL particles has mainly cholesterol esters and small amounts of triglycerides and other lipid molecules. Previous research on analysis of lipids in human atherosclerotic plaques has found evidence about the presence of cholesteryl esters and triglycerides [3]. Cholesteryl esters with polyunsaturated fatty acids were in higher concentration in plaques compared to plasma.

The treatment with statins has shown substantial results in reducing the progression of coronary and carotid atheroma, as measured by intima media thickness, and may also lead to plaque regression [4]. Cholesterol-lowering statin therapy significantly reduces the risk of cardiovascular disease [5,6]. Studies in patients with hypertriglyceridemia have shown that statins can also inhibit triglyceride synthesis [7]. Statins are associated with a decrease in the concentration of the entire set of free fatty acid (FFA) in plasma [8].

Large amounts of plasma FFAs have contributed to an increased risk of cardiovascular disease and mortality [9,10]. In addition, high plasma FFAs levels are associated with ischemic lesion [11] and type 2 diabetes [12]. Recent studies have shown that each FFA has a different behavior in the pathologies of atherosclerosis. Thus, high levels of omega-3 polyunsaturated fatty acids lowered blood pressure [13]. Among saturated fatty acids (FAs) only palmitic acid increased mortality in patients with cardiovascular disease [14]. High levels of trans fatty acids have been associated with inflammation and oxidative stress in coronary heart disease [15]. Many findings suggest the role of long-chain polyunsaturated fatty acids belonging to n-3 and n-6 series in the membrane modulation, eicosanoid metabolism, gene expression, and cellular signaling [16]. These results show the need to study each FA or class of FAs rather than analyze the entire set of FAs together.

Out of seven commercial statins [17], only for simvastatin [8], atorvastatin [8], and rosuvastatin [18], the effect on the concentration of the entire set of FFAs was controlled. The changes in the composition of FFAs [19] and total FAs [20] were evaluated only for simvastatin treatment. In these studies, the mechanism of action of statins on plasma composition of and serum FAs has not been elucidated [8,20]. The effect of rosuvastatin treatment on the concentration of each FA in the fraction of FFAs and total FAs in plasma has not yet been studied.

This study focuses on changing the concentration of each FA in the fraction of FFA and total lipids in human plasma after short-term treatment with rosuvastatin as a cholesterol-lowering drug. We assess the role of cholesterol homeostasis in changing the concentration of plasma FAs.

## 2 Materials and methods

### 2.1 Subjects

The subjects in this study consisted of six Caucasian men over the age of 65 with isolated hypercholesterolemia, defined as total plasma cholesterol between 5.18 and 7.77 mmol/L (200–300 mg/dL), low-density lipoprotein (LDL)-cholesterol above 3,63 mmol/L (140 mg/dL) and triglycerides below 1.13 mmol/L (100 mg/dL). We excluded subjects with any form of cardiovascular disease, diabetes, renal or hepatic diseases, thyroids diseases, autoimmune disorders, smoking, and inflammatory diseases. The subjects did not take medication or supplements during this period.

### 2.2 Study design and clinical information

The study was conducted in accordance with the ethical guidelines on human experimentation developed for the medical community in the Helsinki Declaration and was approved by the Research Ethics Committee of the University of Medicine and Pharmacy of Timisoara. All included subjects gave an informal written consent before participating in this study.

This study had a single group of subjects and began with a 7-day habitual diet in order to stabilize study parameters. Subjects were randomized to receive the first treatment with placebo or rosuvastatin for 4 weeks and after 2 weeks of washout period, they received another medication (placebo or rosuvastatin) for another period of 4 weeks. Rosuvastatin was administrated orally every day at bedtime in a single dose of 20 mg without any change in dosage in this study period. The participating subjects were educated about lifestyle imposed during this study and they agreed to respect the lifestyle modification, regarding diet and exercise. They all ate the same habitual diet throughout the study period.

The study started with a questionnaire about personal medical histories and a comprehensive physical examination, including age, sex, and vascular risk factors such as hypertension, hypocholesteremia, cardiovascular disease, diabetes, etc. The process was placebo-controlled, double-blind, randomized, and crossover study. The subjects, data collectors, and outcome analysts were all unaware of the treatment received by subjects.

### 2.3 Laboratory analyses

The first blood samples analyses were done after a run-in period of 7 days of habitual diet (baseline) and then at the end of the placebo and statin treatment. Blood samples for laboratory analyses were collected at room temperature into commercially available anticoagulant-treated tubes by antecubital venipuncture after an overnight fast of more than 10 h and were immediately centrifuged at 2500×*g* for 14–16 min. The pool plasma samples were divided into aliquots, which were then stored at −70 °C until analysis.

Total cholesterol, triglycerides, LDL-cholesterol, high-density lipoprotein (HDL)-cholesterol, total FAs, and total FFAs were assayed by routine laboratory techniques using standard enzymatic-spectrophotometric methods from Roche Diagnostics (Basel, Switzerland). Phospholipids were assayed by standard enzymatic-spectrophotometric method with a commercial kit available from Wako Diagnostics (Osaka, Japan). Apolipoprotein A-1 and apolipoprotein B-100 were determined by a nephelometric turbidity method using a commercial Siemens ProSpec Analyzer (Siemens Co., Marburg, Germany). Individual FA analysis was performed by gas chromatography-mass spectrometry of their corresponding fatty acid methyl ester. All FAs from plasma lipids were methylated in 15 min at room temperature with methyl iodide in solution of dimethyl sulfoxide and by addition of solid sodium hydroxide [21]. FFAs were selectively methylated in 1 min at room temperature with methyl iodide in a solution of dimethyl sulfoxide and in the presence of anhydrous potassium carbonate [22]. FAs methyl esters were identified by mass spectrometry and on the basis of retention times of the FAs standards. Quantitative evaluation was done using tridecanoic acid as internal standard. The absolute concentration of each fatty acid represents the amount (μmol) of fatty acid contained in 1 L of plasma. All gas chromatography-mass spectrometry analyses of FAs methyl esters were carried out with a gas chromatograph, model Varian 450 GC, coupled with a mass spectrometer, model 240 MS Ion Trap (Agilent, CA, USA).

### 2.4 Statistical analysis

The variables of the statistical analysis measured in this study are the level of total cholesterol, LDL-cholesterol, HDL-cholesterol, triglycerides, phospholipids, apolipoprotein A-1, apolipoprotein B-100, total FAs, total FFAs, and the individual concentration of the FAs from total plasma lipids and plasma FFAs. The observed values of these variables are expressed numerically so that the study data could be statistically analyzed. For these normally distributed variables, the values are expressed as mean ± standard deviation (SD). We have pairs of observed values: one before and another after treatment. Student's paired *t*-test was used for the analysis of the differences in means of the variables within the same treatment group. The differences are explained by random variation. All statistical analyses were two-tailed test and the confidence interval was 95% for the mean changes. The *p*-values greater than the chosen significance level (0.05) indicates that no effect was observed statistically. Microsoft Excel 2016 (Microsoft Corp. USA) and Statistical Test Calculator online version 2018 from Social Science Statistics (https://www.socscistatistics.com/tests/) were used for these statistical analyses.

## 3 Results

Rosuvastatin (20 mg) was well tolerated and none of the side effects shown in the rosuvastatin package leaflet were observed in this short-term treatment. It was possible to select a short period of treatment because it is known that the change in LDL-cholesterol concentration reached a steady state within two weeks of rosuvastatin treatment [23]. The washout period was two weeks because this period was found to be sufficient to eliminate the effect of a previous dose of rosuvastatin up to 40 mg [24]. Table 1 contains the concentrations of the lipid variables. Selected subjects exhibited a high baseline plasma levels of LDL-cholesterol, and total cholesterol and a normal level of triglycerides. Changes in the concentration of lipid variables between baseline and placebo were not significant. Short-term treatment with rosuvastatin significantly decreased total cholesterol, LDL-cholesterol, apolipoprotein B-100, triglycerides, phospholipids, total fatty acids, and total free fatty acids and significantly increased HDL-cholesterol and apolipoprotein A-1. The reduction of total FFAs is very close to that of triglycerides.

**Table 1:** The effect of placebo and rosuvastatin treatment on the concentration of the main variables in this study.

Variable	Absolute concentration (mean ± SD)			Difference PB – BL (% mean change)	Difference RS – BL (% mean change)
	Baseline (BL)	Placebo (PB)	Rosuvastatin (RS)		
Total cholesterol, mmol/L	6.17 ± 0.47	6.16 ± 0.44	3.81 ± 0.33	−0.01 ± 0.06^a^ (−0.16)	−2.36 ± 0.14^e^ (−38.25)
LDL-cholesterol, mmol/L	4.57 ± 0.32	4.56 ± 0.30	2.18 ± 0.19	0.01 ± 0.06^a^ (0.22)	−2.39 ± 0.13^e^ (−52.29)
HDL-cholesterol, mmol/L	1.175 ± 0.15	1.186 ± 0.14	1.273 ± 0.16	0.011 ± 0.02^a^ (0.93)	0.098 ± 0.02^b^ (8.34)
Apolipoprotein B-100, nmol/L	322.34 ± 46.91	321.90 ± 47.52	175.02 ± 19.15	−0.44 ± 4.54^a^ (−0.13)	−147.31 ± 31.24^c^ (−45.70)
Apolipoprotein A-1, nmol/L	37.11 ± 3.44	37.17 ± 3.50	39.17 ± 3.47	0.05 ± 0.28^a^ (0.13)	2.06 ± 0.14^e^ (5.55)
Triglycerides, mmol/L	0.93 ± 0.078	0.94 ± 0.063	0.74 ± 0.065	0.01 ± 0.03^a^ (1.08)	−0.19 ± 0.02^d^ (−20.43)
Phospholipids, mmol/L	2.45 ± 0.10	2.46 ± 0.12	1.78 ± 0.05	0.01 ± 0.03^a^ (0.41)	−0.67 ± 0.06^d^ (−27.35)
Total fatty acids, mmol/L	13.11 ± 0.54	13.17 ± 0.50	9.16 ± 0.28	0.06 ± 0.22^a^ (−0.46)	−3.95 ± 0.29^e^ (−30.13)
Total free fatty acids, μmol/L	683.55 ± 29.58	682.06 ± 26.17	537.22 ± 18.36	−1.49 ± 9.54^a^ (−2.18)	−146.33 ± 18.12^d^ (−21.41)

^a^*p* > 0.05; ^b^*p* < 0.001; ^c^*p* < 0.0001; ^d^*p* < 0.00001; ^e^*p* < 0.000001.

The FAs in plasma could be non-esterified in FFAs and esterified in triglycerides, cholesteryl esters, and phospholipids. One molecule of triglyceride has three fatty acid molecules in its structure, while one molecule of cholesteryl ester has one molecule of fatty acid. Cholesteryl esters represent approximately 70% of the total cholesterol [25]. The main components of the phospholipids are phosphatidylcholine (70.4%), lysophosphatidylcholine (6.2%), and sphingomyelin (17.4%) [25]. They represent approximately 94% of the phospholipids and contain a phosphocholine group which is basic in enzymatic analysis. The other 6% represent mainly phosphatidylethanolamine, phosphatidylserine, and phosphatidylinositol. Each phospholipid component has two fatty acid molecules in their chemical structure, with two exceptions: lysophosphatidylcholine and sphingomyelin, which have a single fatty acid molecule. Taking into account the structure of these compounds and their concentration in the plasma lipids, the concentration of total FAs at baseline was 12.56 mmol/L, which is comparable with the one that was determined experimentally.

Table 2, Table 3 show the absolute concentrations at baseline and at the end of rosuvastatin treatment for each FA in the total plasma lipids and the FFA fraction, respectively. The difference in absolute concentrations between rosuvastatin treatment and baseline reflects the effect of rosuvastatin treatment on each FAs. The differences in absolute concentrations between placebo and baseline were not-significant and were therefore not introduced in Table 2, Table 3 The FAs traces add up all the small amounts of FAs that were not included in these tables.

**Table 2:** The effect of rosuvastatin treatment on the concentration of each fatty acid in total plasma.

Fatty acid	Absolute concentration^a^, μmol/L (Relative concentration, %)		Change from baseline (% mean change)	Effect, *p* value
	Baseline	Rosuvastatin		
Dodecanoic acid (C12:0)	127.17 ± 7.52 (0.97)	64.85 ± 6.75 (0.71)	−62.33 ± 5.58 (−49.01)	<0.00001
Tetradecanoic acid (C14:0)	302.85 ± 29.43 (2.31)	153.92 ± 11.45 (1.68)	−148.93 ± 19.64 (−49.18)	<0.00001
Pentadecanoic acid (C15:0)	43.26 ± 2.40 (0.33)	40.57 ± 4.49 (0.44)	−2.69 ± 2.23 (−6.23)	<0.05
Hexadecanoic acid (C16:0)	2677.12 ± 200.2 (20.42)	1365.59 ± 187.43 (14.91)	−1311.53 ± 101.87 (−48.99)	<0.00001
Heptadecanoic acid (C17:0)	85.22 ± 4.29 (0.65)	80.65 ± 5.12 (0.88)	−4.57 ± 2.32 (−5.36)	<0.01
Octadecanoic acid (C18:0)	1162.88 ± 95.66 (8.87)	614.02 ± 41.55 (6.70)	−548.86 ± 68.66 (−47.20)	<0.00001
Eicosanoic acid (C20:0)	44.58 ± 2.52 (0.34)	39.76 ± 3.43 (0.43)	−4.81 ± 1.57 (−10.80)	<0.001
Docosanoicacid (C22:0)	111.44 ± 7.4 (0.85)	98.11 ± 11.49 (1.07)	−13.33 ± 4.43 (−11.96)	<0.001
Hexadecenoic acid (C16:1) n-7	283.18 ± 15.4 (2.16)	253.18 ± 23.41 (2.76)	−30.00 ± 9.72 (−10.59)	<0.001
Octadecenoic acid (c-C18:1) n-9	3265.78 ± 264.06 (24.91)	1833.63 ± 175.47 (20.2)	−1432.17 ± 160.47 (−43.85)	<0.00001
Eicosenoic acid (C20:1) n-9	85.22 ± 5.90 (0.65)	76.34 ± 8.61 (0.83)	−8.68 ± 2.57 (−10.42)	<0.001
Octadecadienoic acid (C18:2) n-6	2833.14 ± 192.36 (21.61)	2425.03 ± 265.55 (26.48)	−408.11 ± 79.14 (−14.40)	<0.0001
Eicosadienoic acid (C20:2) n-9	32.78 ± 4.17 (0.25)	33.08 ± 3.09 (0.36)	0.30 ± 1.49 (0.93)	0.65
Octadecatrienoic acid (C18:3) n-3	74.73 ± 5.38 (0.57)	70.05 ± 5.71 (0.76)	−4.68 ± 1.10 (−6.26)	<0.01
Eicosatrienoic acid (C20:3) n-6	166.50 ± 11.27 (1.27)	170.57 ± 12.05 (1.86)	4.07 ± 1.65 (2.44)	<0.01
Eicosatetraenoic acid (C20:4) n-6	978.03 ± 82.0 (7.46)	1005.83 ± 78.26 (10.98)	27.80 ± 7.40 (2.84)	<0.001
Docosatetraenoic acid (C22:4) n-6	89.15 ± 11.69 (0.68)	91.48 ± 10.44 (1.00)	2.33 ± 1.64 (2.61)	<0.05
Docosapentenoic acid (C22:5) n-3	142.90 ± 11.21 (1.09)	143.86 ± 11.66 (1.57)	0.96 ± 0.61 (0.67)	<0.05
Docosahexaenoic acid (C22:6) n-3	268.76 ± 18.8 (2.05)	271.81 ± 18.53 (2.97)	3.05 ± 1.35 (1.13)	<0.01
FA trace	335.62 ± 20.77 (2.56)	325.49 ± 23.44 (3.55)	−10.13 ± 5.19 (−3.02)	<0.01

a Mean ± SD.

**Table 3:** The effect of rosuvastatin treatment on the individual concentration of free fatty acids in plasma.

Free fatty acid	Absolute concentration^a^, μmol/L (Relative concentration, %)		Change from baseline (% mean change)	Effect, *p* value
	Baseline	Rosuvastatin		
Dodecanoic acid (C12:0)	15.74 ± 0.82 (2.32)	12.33 ± 1.24 (2.30)	−3.41 ± 0.67 (−21.66)	<0.0001
Tetradecanoic acid (C14:0)	29.12 ± 2.05 (4.26)	22.67 ± 3.03 (4.22)	−6.45 ± 1.22 (−22.15)	<0.0001
Pentadecanoic acid (C15:0)	5.33 ± 0.49 (0.78)	4.21 ± 0.41 (0.78)	−1.12 ± 0.42 (−21.04)	<0.01
Hexadecanoic acid (C16:0)	170.96 ± 10.1 (25.01)	131.40 ± 14.14 (24.46)	−39.56 ± 4.87 (−23.14)	<0.00001
Heptadecanoic acid (C17:0)	10.39 ± 0.78 (1.52)	8.36 ± 1.17 (1.56)	−2.03 ± 0.56 (−19.54)	<0.001
Octadecanoic acid (C18:0)	68.56 ± 4.11 (10.03)	53.24 ± 3.93 (9.91)	−15.32 ± 1.96 (−22.35)	<0.00001
Eicosanoic acid (C20:0)	3.76 ± 0.17 (0.55)	2.89 ± 0.63 (0.54)	−0.87 ± 0.51 (−23.13)	<0.01
Docosanoicacid (C22:0)	7.93 ± 0.51 (1.16)	6.18 ± 0.75 (1.15)	−1.75 ± 0.37 (−22.06)	<0.001
Hexadecenoic acid (C16:1) n-7	21.46 ± 1.44 (3.14)	16.83 ± 2.01 (3.13)	−4.63 ± 0.78 (−21.59)	<0.0001
Octadecenoic acid (c-C18:1) n-9	228.44 ± 12.71 (33.42)	178.51 ± 17.04 (33.23)	−49.93 ± 5.49 (−21.86)	<0.00001
Eicosenoic acid (C20:1) n-9	5.61 ± 0.24 (0.82)	4.36 ± 0.66 (0.81)	−1.25 ± 0.44 (−22.21)	<0.01
Octadecadienoic acid (C18:2) n-6	92.60 ± 4.34 (13.53)	73.71 ± 5.02 (13.72)	−18.89 ± 2.03 (−20.40)	<0.00001
Eicosadienoic acid (C20:2) n-9	0.75 ± 0.02 (0.11)	0.73 ± 0.12 (0.14)	−0.02 ± 0.10 (−2.91)	0.62
Octadecatrienoic acid (C18:3) n-3	1.51 ± 0.20 (0.22)	1.19 ± 0.15 (0.22)	−0.32 ± 0.12 (−21.19)	<0.01
Eicosatrienoic acid (C20:3) n-6	1.03 ± 0.04 (0.15)	1.01 ± 0.15 (0.19)	−0.02 ± 0.12 (−1.49)	0.69
Eicosatetraenoic acid (C20:4) n-6	9.02 ± 0.49 (1.32)	9.15 ± 0.69 (1.70)	0.13 ± 0.22 (1.41)	0.20
Docosatetraenoic acid (C22:4) n-6	1.50 ± 0.07 (0.22)	1.48 ± 0.28 (0.28)	−0.02 ± 0.22 (−1.58)	0.83
Docosapentenoic acid (C22:5) n-3	0.89 ± 0.04 (0.13)	0.87 ± 0.01 (0.16)	−0.02 ± 0.07 (−2.09)	0.64
Docosahexaenoic acid (C22:6) n-3	1.64 ± 0.06 (0.24)	1.67 ± 0.16 (0.31)	0.03 ± 0.12 (1.80)	0.58
FA trace	7.31 ± 0.21 (1.07)	6.43 ± 0.85 (1.20)	−0.88 ± 0.66 (−12.09)	0.023

a Mean ± SD.

Table 2 shows that the absolute concentrations of saturated FAs in total FAs were strongly affected by rosuvastatin, with a significant decrease of approximately 48%. Monounsaturated FAs had reductions around 10%. Octadecenoic acid n-9 (C18:1) was an exception with a decrease of 43.85%. Long-chain polyunsaturated fatty acids had small but significant increases. Octadecadienoic acid n-6 (C18:2) was an exception with a significant decrease close to monounsaturated FAs.

Table 3 shows that, compared to baseline, rosuvastatin treatment significantly reduced the absolute concentrations of saturated and monounsaturated FFAs. Their changes are close to those of triglycerides. The series of polyunsaturated FFAs synthetized from linoleic and linolenic acids had no significant change. The relative concentrations of plasma FFAs obtained with rosuvastatin treatment compared to baseline remained relatively constant.

## 4 Discussion

Rosuvastatin can inhibit hydroxymethylglutaryl-coenzyme A reductase in the cholesterol biosynthesis. Rosuvastatin is superior to all other statins in lowering LDL-cholesterol [26].

Cholesterol and FAs have multifunctional roles in the human body. Blood cholesterol and FAs are obtained by biosynthesis from less complex substances inside the body and can also be obtained from food by absorption in the gastrointestinal tract. In the particles of LDL-cholesterol, the nonpolar lipids such as cholesteryl ester and triglyceride represent a nonpolar core that is surrounded by a coating, consisting of phospholipids, apolipoproteins, free cholesterol, and small amounts of free fatty acids. These complex particles of lipoproteins are the main vehicle for the circulation of lipids in the blood, being divided into four categories according to their densities. These lipoprotein particles have different concentrations of lipids. From the content of total lipid in LDL particle, the apolar core contains approximately 50% cholesteryl esters and 10% triglycerides. The LDL core is surrounded by a unilamellar surface containing mainly free cholesterol (10%), phospholipids (30%) and apolipoproteins [27]. The content of triglycerides in chylomicrons, very low-density lipoproteins, and LDL is inversely proportional to the content of cholesterol and phospholipids. The main role of LDL is to transport cholesterol in blood to non-hepatic tissues (peripheral cells). HDL is a lipoprotein particle used for the cholesterol transport from peripheral tissues to the liver.

Cholesterol concentration is closely adjusted in a dynamic state of equilibrium through a feedback control system that operates at transcriptional and posttranscriptional levels [28]. This control of cholesterol concentration is achieved through a variety of mechanisms [29], such as biosynthesis, intestinal absorption, uptake through LDL receptors, transport to peripheral cells by LDL, reverse cholesterol transport to the liver for recycling and degradation, storage by esterification, and conversion into bile acids. These homeostatic mechanisms are only partly understood.

Due to cholesterol homeostasis, the inhibition of the biosynthesis of cholesterol by rosuvastatin will induce a feedback effect with many regulatory mechanisms in order to reach the equilibrium state, reducing the amount of cholesterol transported to peripheral cells by LDL, increasing the reverse cholesterol transport with HDL, reducing the hepatic cholesterol catabolism and increasing the intestinal cholesterol absorption. The decrease in LDL-cholesterol levels in our case by 52.29% using rosuvastatin treatment means that the number of LDL particles was significantly reduced. As a consequence, all components that enter into the structure of LDL particles, such as fatty acids, triglycerides, phospholipids, and apolipoprotein B-100, suffered a significant decrease. The decrease in the levels of fatty acids, triglycerides, phospholipids, and apolipoprotein B-100 is not the result of a direct involvement of rosuvastatin in the synthesis of these compounds, but rather of some feedback regulatory mechanisms generated by the direct action of rosuvastatin in the stage of cholesterol biosynthesis.

Because cholesterol biosynthesis is partially blocked by rosuvastatin, regulatory mechanisms will act to recover the cholesterol from the peripheral cells, which requires an increase in HDL-cholesterol and implicitly an increase in the production of apolipoprotein A. These regulatory mechanisms are obviously a reason for elevated levels of HDL-cholesterol and apolipoprotein A-1. This efflux of peripheral cholesterol to HDL and its transport to the liver is important for the anti-atherogenic properties of HDL.

FAs are the main components in the structure of triglycerides, phospholipids, and cholesteryl esters, and therefore a decrease in their plasma concentration by inhibition the cholesterol synthesis with rosuvastatin automatically means a decrease in the concentration of total FAs. Practically, it is a strong coordinated feedback control of hepatic cholesterol and FAs biosynthesis, because the transport of hepatic FAs to the peripheral tissues and cells can be done together with cholesterol as complex particles of lipoprotein. Negative feedback mechanisms will act to reduce both biosynthesis and intestinal absorption of FAs. These regulatory mechanisms in cholesterol homeostasis appear to be the main reason for the change in the concentration of each FA in total lipid FAs and FFAs in our experiments.

The concentration of each FA in plasma lipids is given by the sum of its amounts in each lipid molecule. The magnitude of the change in the concentration of each FA caused by rosuvastatin treatment is the sum of the changes in their concentration in all lipids and also depends on the mechanisms of homeostatic regulation. In Table 2, it can be seen that all quantitatively significant FAs had very significant reductions and FAs with lower concentrations had less significant reductions. There are some exceptions given by long-chain polyunsaturated fatty acids, which are obtained by elongation and desaturation of essential acids: linolenic acid (C18:3) and linoleic acid (C18:2). These two essential FAs and other saturated and unsaturated FAs were obtained in this experiment by intestinal absorption, mostly from sunflower oil (65% C18:2, 0.1% C18:3) and pig meat (13% C18:2, 0.9% C18:3) [30]. We assume that the small increase in long-chain polyunsaturated fatty acids is also the result of the mechanisms of cholesterol homeostasis that increase the reverse transport of cholesterol with HDL particles. The reverse cholesterol transport involves the transformation of cellular free cholesterol to its insoluble ester in the presence of lecithin cholesterol acyl transferase [31]. This enzyme is put into circulation by the liver [27]. FAs used to esterify free cholesterol are taken from phospholipids located on the surface of HDL particles. Long-chain polyunsaturated fatty acids are mainly used in this esterification process, because the composition of FAs in HDL-cholesteryl esters has over 80% long-chain polyunsaturated fatty acids [32]. The increase in HDL-cholesterol level using cholesterol homeostasis mechanisms may thus explain the smaller decrease in linoleic and linolenic acids compared to saturated acids and the small increase in long-chain polyunsaturated fatty acids synthetized from linoleic and linolenic acid, such as C20:4 (n-6) and C22:6 (n-3), respectively.

Circulating FFAs are mainly obtained from triglycerides by the action of lipoprotein lipases [27]. It is known that the composition of FAs in the fraction of FFAs is very similar to that of human plasma triglycerides [33]. Thus, it is no surprise that the decrease in each FA concentration in FFAs is very close to that of triglycerides. Only polyunsaturated fatty acids are an exception. Their small concentrations are practically unchanged and this could be caused by their small increase in total lipid FAs, which may be associated with the increase of the HDL-cholesterol level.

The new finding that the concentration of polyunsaturated fatty acids increased after rosuvastatin treatment may have beneficial consequences because a high level of n-3 polyunsaturated FAs lowered blood pressure [13]. Polyunsaturated fatty acids may also be beneficial for many inflammatory disorders associated with cardiovascular diseases, obesity, arthritis, and asthma [16]. The finding that the concentration of saturated FAs decreased after rosuvastatin treatment may have beneficial effects, because the reduction in plasma palmitic acid has been associated with a low mortality in patients with cardiovascular disease [14]. The finding that rosuvastatin decreased total plasma FFAs may be beneficial in cardiovascular diseases [9,10] and type 2 diabetes [12].

This is a preliminary study that could have some limitations, given the small number of men subjects, the short-term treatment and the use of only one dose of rosuvastatin, which is not at maximal concentration. We used only men in this study because the average age of cardiovascular diseases is 65 for men, while for women these events are observed at ages of 73–75 years [34]. Despite our small number of subjects, we employed a tightly designed crossover study, placebo-controlled, double-blind, and randomized. The possible limitation generated by the number of subjects was reduced by using a crossover experiment with two treatment periods [35] and a homogeneous group of Caucasian men. This study should be analyzed with these limitations and cannot be considered to have general applicability. The observed changes were significant. Further studies should include the administration of different doses of rosuvastatin.

## 5 Conclusions

In conclusion, it can be assumed that by blocking the biosynthesis of cholesterol by rosuvastatin, the regulatory mechanisms in cholesterol homeostasis will try to maintain the equilibrium with less cholesterol, changing the concentrations of lipids involved in cholesterol homeostasis. Rosuvastatin is directly involved only in the biosynthesis of cholesterol and indirectly in the biosynthesis of apolipoprotein A-1 and B-100, fatty acids, triglycerides, phospholipids, and cholesteryl esters. The role of cholesterol homeostasis is not exclusive, but is supported by observations, and suggests that cholesterol homeostasis through its regulatory mechanisms is the main cause of changes in the concentration of each plasma fatty acid during rosuvastatin treatment. Rosuvastatin treatment significantly decreased the absolute concentrations of saturated and monounsaturated FAs in the FFA fraction, as well as in the total FAs. Long chain polyunsaturated fatty acids with 20 and 22 carbon atoms in the molecule had no significant change in the fraction of FFAs. The effects of rosuvastatin treatment on the concentration of each fatty acid in the fraction of FFAs and total plasma lipids may be a source of beneficial consequences, in addition to lowering LDL-cholesterol in cardiovascular diseases. Our findings provide an explanation of the changes in the concentration of each plasma fatty acid based on the mechanisms of cholesterol homeostasis.

## CRediT authorship contribution statement

**Cristian I. Ciucanu:** Conceptualization, Methodology, Software, Validation, Formal analysis, Investigation, Writing - original draft, Writing - review & editing. **Sonia Olariu:** Writing - original draft, Investigation, Statistical analysis, Interpretation of data, and Drafted the manuscript. **Daliborca C. Vlad:** Investigation, Performed the analysis, Interpretation of data. **Victor Dumitraşcu:** Validation, Supervition, All authors read and approved the final version.

## Declaration of competing interest

The authors declare that they have no known competing financial interests or personal relationships that could have appeared to influence the work reported in this paper.
